# Mlp4green: A Binary Classification Approach Specifically for Green Odor

**DOI:** 10.3390/ijms25063515

**Published:** 2024-03-20

**Authors:** Jiuliang Yang, Zhiming Qian, Yi He, Minghao Liu, Wannan Li, Weiwei Han

**Affiliations:** Key Laboratory for Molecular Enzymology and Engineering of Ministry of Education, School of Life Sciences, Jilin University, Changchun 130012, China; jiuliang23@mails.jlu.edu.cn (J.Y.); qianzm1321@mails.jlu.edu.cn (Z.Q.); heyi21@mails.jlu.edu.cn (Y.H.); lmh23@mails.jlu.edu.cn (M.L.)

**Keywords:** machine learning, green odor, molecular docking, odor prediction, binary classification

## Abstract

Fresh green leaves give off a smell known as “green odor.” It has antibacterial qualities and can be used to attract or repel insects. However, a common method for evaluating green odor molecules has never existed. Machine learning techniques are widely used in research to forecast molecular attributes for binary classification. In this work, the green odor molecules were first trained and learned using machine learning methods, and then clustering analysis and molecular docking were performed to further explore their molecular characteristics and mechanisms of action. For comparison, four algorithmic models were employed, MLP performed the best in all metrics, including Accuracy, Precision, Average Precision, Matthews coefficient, and Area under curve. We determined by difference analysis that, in comparison to non-green odor molecules, green odor molecules have a lower molecular mass and fewer electrons. Based on the MLP algorithm, we constructed a binary classification prediction website for green odors. The first application of deep learning techniques to the study of green odor molecules can be seen as a signal of a new era in which green odor research has advanced into intelligence and standardization.

## 1. Introduction

Fresh green leaves release an odor known as “green odor” [1]. The “green odor” comes mainly from eight volatile c6-aromatic compounds: 3Z-Hexenol, 3E-Hexenol, 2E-Hexenol, 3Z-Hexenal, 3E-Hexenal, 2E-Hexenal, n-Hexanol, and n-Hexanal [2]. Studies have shown that green scents can relieve the body’s tiredness and have a soothing and calming effect on the nervous system. The green odor relieves depressive conditions, eases feelings of fatigue, and attenuates stress in humans and mice [3,4,5,6,7,8,9,10]. Green odor also has a wide range of biological effects. For example, one study revealed its antimicrobial effects against two Gram-positive and three Gram-negative strains of bacteria [11]. In addition to having certain fungicidal qualities, green scents can be utilized to draw or repel insects [2]. Certain insects consume green leaves, which introduces green odor molecules into their bodies. These chemicals are then used by the insects as pheromones for functions like communication [9]. Green odors have been studied as far back as a few decades ago. As a class of functional compounds, green odorants have the potential to serve as a library of new candidate compounds for antidepressants, antimicrobials, and more. The discovery of green odor compounds can serve as a basis for new discoveries in drug screening. The predictive study of green odor compounds is novel and significant.

Plants have a complex composition of volatile odor compounds, of which green odorants are difficult to identify. Prediction by wet experiment methods is time-consuming and laborious. Prediction using machine learning methods can compensate for these shortcomings. In this study, for the first time, a machine learning method was used to predict whether a molecule has a green odor. The predictive performance of machine learning models can be judged by recognized metrics such as Acc (Accuracy), Pre (Precision), AP (Average Precision), MCC (Matthews coefficient), and AUC (Area Under Curve). The higher the above metrics are, the more accurate the model predictions are. Prediction of molecular properties using machine learning is both simple and efficient. By learning the structural features of molecules, it is possible to binary classify molecules of a particular nature with high accuracy. Therefore, this study hypothesizes that green odor molecules have potentially common structural features that can be learned by machine learning methods, which in turn can be predicted with high accuracy for unknown molecules. With the results of machine learning, the study will further explore the chemical structure and mechanism of action of green odor molecules, revealing the potential laws of their functioning. Additionally, by using an internet service that is freely available to everyone, the binary classifiers developed through the application of machine learning can be employed, saving time and money [12,13,14,15,16,17]. This study presents a new breakthrough in the field of green odor research by applying machine learning for the first time to green odor molecular prediction.

This study compares the various evaluation metrics of MLP, SVM, RF, and MPNN algorithms in determining whether a molecule has a green odor. We chose the best performing MLP algorithm for designing the prediction program and building a user-actionable website. Using molecular fingerprint similarity and chemical spatial network distances, we clustered green odor molecules. To evaluate chemical attributes and describe trends, we performed a difference analysis on molecules located in distinct clusters. We carried out a molecular docking investigation to gain further insight into the molecular mechanism underlying the formation of green odor. The *Anopheles gambiae*’s odor receptor [18,19] was chosen as the study’s target protein, and significant residues interacting with representative molecules from other clusters were compared. Research on bactericidal and insect properties heavily relies on the ability to identify chemicals that smell green. Prospective green odorants could be applied to forest preservation and agricultural production as fungicidal repellents [20]. The identification of more green odorant-molecules will greatly advance the study of insects, as green odorants have the potential to function as insect pheromones.

## 2. Results

### 2.1. Cluster Analysis

**Figure 1:**
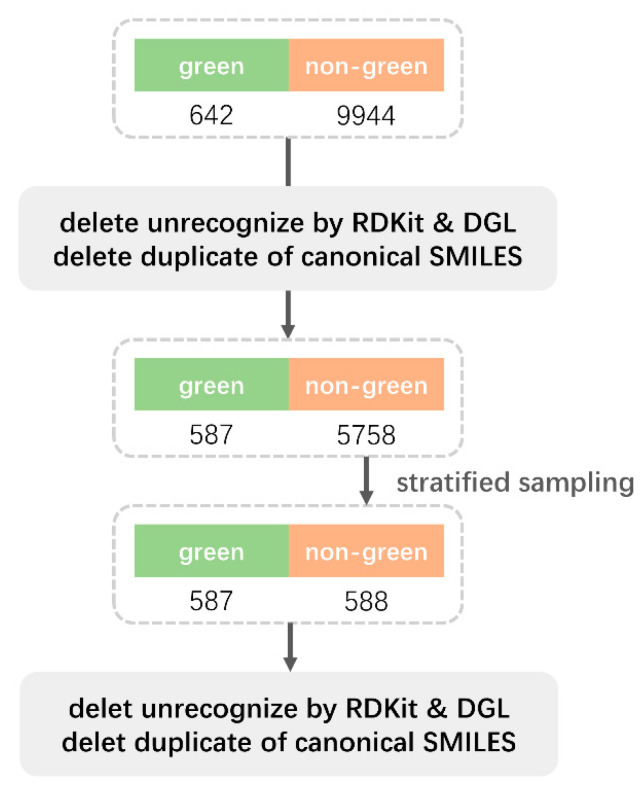
Dataset Processing Flowchart.

**Figure 2:**
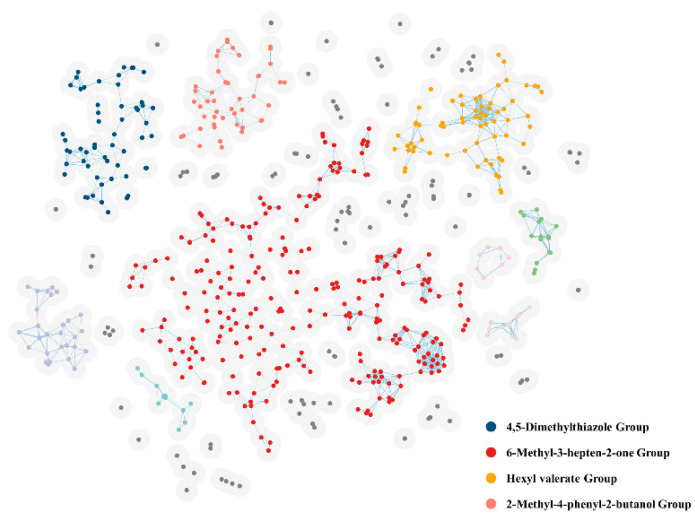
Cluster analysis of 587 green odor molecules. The light gray circles represent the threshold radii of the molecules, and molecules with two intersecting radii are divided into groups; Groups with less than 7 molecules are shown in gray, and groups with more than 7 molecules are shown in color. Groups of different molecules are shown in different colors.

Following data cleaning, we were left with 587 molecules of green odor (Figure 1). On these chemicals causing the green odor, we performed a cluster analysis. These 587 molecules were divided into various groups, as shown in Figure 2. Out of the 587 molecules, 441 molecules fell into four groups after the groups containing fewer than 30 molecules were removed. Fingerprint similarity was calculated using the Dice coefficient. We selected the molecules with the highest similarity in each of the four groups as representative molecules by similarity calculation (Table 1). Each group was named after a representative molecule. These groups are 2_Methyl_4_phenyl_2_butanol group, 4,5-Dimethylthiazolel group, 6-Methyl-3-hepten-2-one group, and Hexyl valerate group.

### 2.2. Docking Study

#### 2.2.1. 2_Methyl_4_phenyl_2_butanol Group

**Figure 3:**
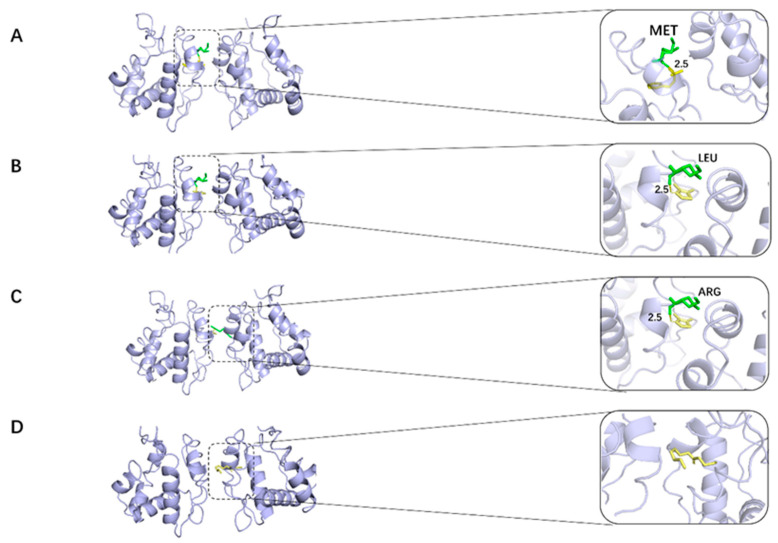
Results of the molecular docking analysis of four typical molecules for the insect odor-binding protein (OBP). (**A**): 2_Methyl_4_phenyl_2_butanol docked to the active residues around OBP. (**B**): Dimethylthiazolel docked to the active residues around OBP. (**C**): 6-Methyl-3-hepten-2-one docked to the active residues surrounding OBP. (**D**): Hexyl valerate docked to the active residues around OBP. (The yellow portion is the small molecule used for docking and the green portion is the amino acid residue where the small molecule creates an interaction).

The interaction of MET89 and 2_methyl_4_phenyl_2_butanol with the protein receptor OBD is depicted in Figure 3A. The interaction between MET89 and the protein receptor is represented by the yellow dashed line, which is hydrogen bonding. The active amino acid residues that bind to the target proteins are MET89, LEU73, TRP114, LEU76, and ALA88, and the compounds form stronger hydrogen bonds with the active group of MET89 at a distance of 2.5 Å, which is smaller than the 3.5 Å of conventional hydrogen bonds, which is a stronger bond and plays an important role in stabilizing the small molecule ligands. In addition, the benzene ring of the molecule also forms a strong π-π conjugation interaction with amino acid TRP114 of the active pocket of the protein, and there are π-alkyl forces with amino acid residues LEU73, LEU76 and ALA88 of the protein. In addition, there are several amino acids with the compound with van der Waals forces.

#### 2.2.2. 4,5-Dimethylthiazolel Group

Figure 3B demonstrates the docking of 4,5-Dimethylthiazolel with the protein receptor OBD. The compounds establish stronger hydrogen bonds with the active groups of MET89 and LEU73 at a distance of 2.5 Å, which is less than that of the conventional hydrogen bond of 3.5 Å, which is a stronger bond and is crucial for stabilizing the small-molecule ligand. These compounds bind to the target protein 4 through the active amino acid residues MET89, LEU73, and TRP114. The five-membered ring of dimethylthiazole also forms a strong π-alkyl interaction with the protein’s active pocket amino acid TRP114. Additionally, the compound has an alkyl force with the protein’s residue LEU73. Several amino acids also have van der Waals forces with the compound.

#### 2.2.3. 6-Methyl-3-hepten-2-one Group

Methyl_3_hepten_2_one binds to target protein 6 through the active amino acid residues ARG94, HIS77, LEU76, LEU80, ALA88, and TRP114, shown in Figure 3C. The compounds form stronger hydrogen bonds with the ARG94 active group at a distance of 3.0 Å, which is less than the conventional hydrogen bond of 3.5 Å. Methyl_3_hepten_2_one additionally forms van der Waals forces with multiple amino acids and strong π-W alkyl conjugation interactions with amino acids TRP114 and HIS77 of the protein’s active pocket, as well as alkyl interactions with amino acid residues LEU80, LEU76, and ALA88.

#### 2.2.4. Hexyl Valerate Group

LEU96, LEU73, and HIS77 are the active amino acid residues that Hexyl valerate binds to the target protein 6 in Figure 3D. This molecule is small and potentially active, as evidenced by the strong π-alkyl interactions hexyl valerate forms with the HIS77 amino acid of the protein’s active pocket, the alkyl forces it has with the protein’s amino acid residues LEU73 and LEU96, and the van der Waals forces it has with several amino acids in the compounds.

**Figure 4:**
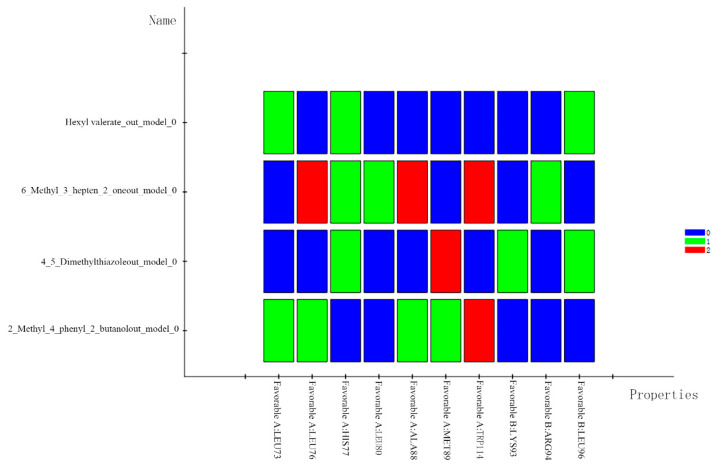
Heat map analysis of molecular docking of four representative molecules.

As above, the molecular docking thermograms were analyzed as shown in Figure 4. For the 6-methyl-3-hepten-2-one subgroup, LEU76, ALA88, and TRP114 generated the highest number of hydrogen bonds with the odorant small molecules, which are the key residues in the docking process. The 4,5-Dimethylthiazolel group and 2_Methyl_4_phenyl_2 group depend on MET89 and TRP114. The interaction between the 4,5-Dimethylthiazolel group and the 2_Methyl_4_phenyl_2_butanol group, respectively, is significantly influenced by MET89 and TRP114.

#### 2.2.5. Quantum Chemical Calculations

**Figure 5:**
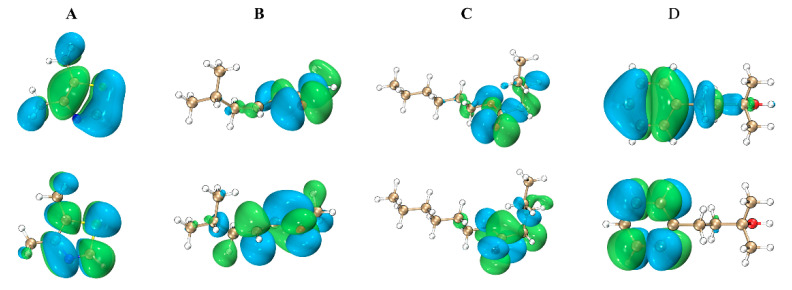
(**A**): HOMO orbit (left) of Dimethylthiazolel; LUMO orbit (right) of Dimethylthiazolel. (**B**): HOMO orbit (left) of 6-Methyl-3-hepten-2-one; LUMO orbit (right) of 6-Methyl-3-hepten-2-one. (**C**): HOMO orbit (left) of Hexyl valerate; LUMO orbit (right) of Hexyl valerate. (**D**): HOMO orbit (left) of 2_Methyl_4_phenyl_2_butanol; LUMO orbit (right) of 2_Methyl_4_phenyl_2_butanol.

The HUMO, LUMO orbital diagrams of four representative molecules are shown in Figure 5.

### 2.3. Evaluate Different Machine Learning Algorithms to Predict Green Odor

**Figure 6:**
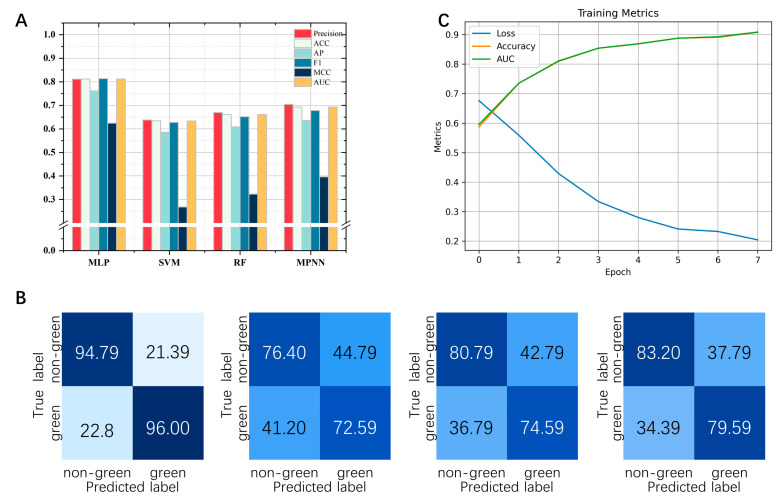
(**A**): Performance of the 4 models on 6 performance metrics. (**B**): Four models’ confusion matrix (MLP, RF, SVM, MPNN). The genuine label of the molecule is represented by the vertical coordinate of the graph, while the anticipated label is represented by the horizontal coordinate. (**C**): Performance of data training in the model. The color shade indicates how high or low the predicted value is for that region.

To evaluate different machine learning algorithms used to predict green odors, this study presents their test results in the form of a confusion matrix (Figure 5B). Cross-validation was performed 5 times and repeated 10 times on the green odor/non-green odor molecular dataset. The labels on the confusion matrix are their average values. In addition, we also compared the evaluation metrics of different algorithms when facing the same dataset, and the specific values are shown in Figure 6A. In summary, the MLP algorithm shows greater advantages compared with other algorithms. We trained the dataset using the MLP algorithm model (Figure 6C).

### 2.4. Factor Analysis

**Figure 7:**
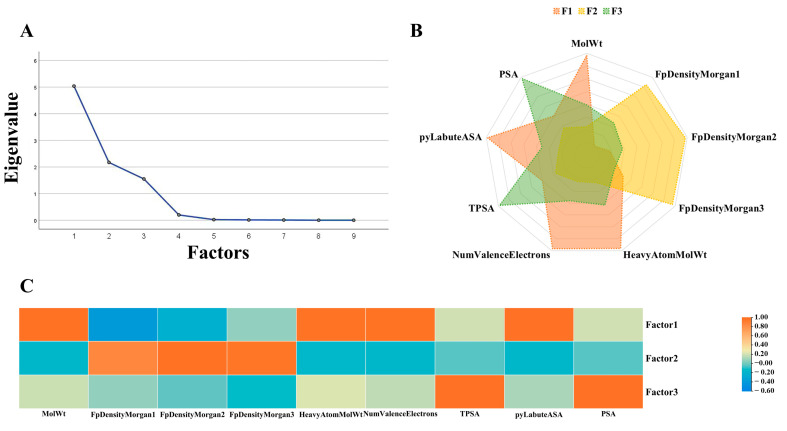
Factor analysis of green and non-green datasets. (**A**): Scree plot of factor eigenvalues. (**B**): Radar plot of the three factors comprising the filtered descriptors. (**C**): Heat map of factor loading matrix.

**Figure 8:**
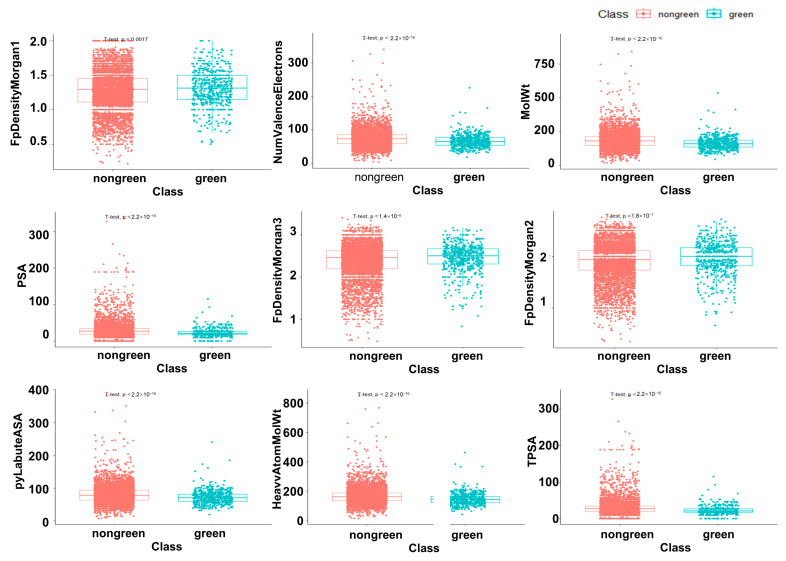
Analysis of significant differences in 9 properties of green and non-green odor molecules.

A total of 208 characteristics of green and non-green odor molecules were factor examined. The high eigenvalues of Factor 1, Factor 2, and Factor 3 are evident in Figure 7. These three elements were our choice for common factors. The factor loadings for Factor 1, Factor 2, and Factor 3 on the nine descriptors (Table 2) are displayed in Figure 7B. Table 2 displays the association between these three common parameters and the nine descriptors; Figure 7C shows that the greater the correlation and the deeper the hue, the larger the value. We examined how the nine characteristics of green and non-green odor molecules differed from one another (Figure 8) when the significance level is below 0.05. The nine metrics are FpDensityMorgan1, FpDensityMorgan2, FpDensityMorgan3, NumValenceElectrons, MolWt, PSA, pyLabuteASA, HeavyAtomMolWt, and TPSA.

### 2.5. The Difference Analysis between Green and Non-Green

The analysis demonstrates that the corresponding features of green odor molecules and non-green odor molecules differ from one another. The *p*-values of green and non-green odor molecules in the nine attributes, as shown in Figure 8, are less than 0.05, suggesting that there is a significant difference between the two groups in the corresponding qualities. Non-green odor molecules have higher PSA and TPSA, while green odor molecules have smaller molecular weights, electron counts, and higher morgan fingerprints 1–3.

### 2.6. Webserver

**Figure 9:**
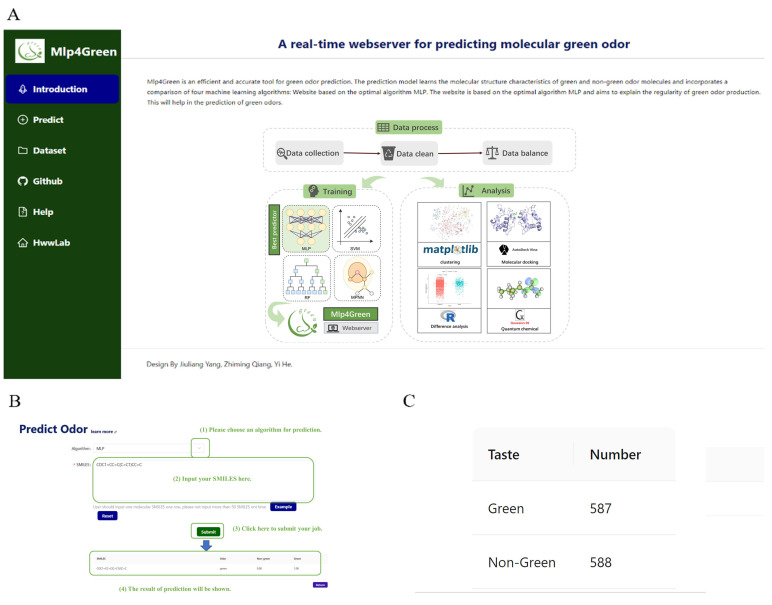
Screenshot of Mlp4green website. (**A**) Home Page and Introduction. (**B**) Tutorials and demos on how to use ourwebsite. (**C**) To show the number of molecules in the training set and to offer to download it in our website.

Based on the selected MLP model, we built a website named Mlp4green with the URL (https://hwwlab.com/webserver/mlp4green, accessed on 20 December 2023). The smiles formula of the molecule is entered on the prediction homepage of the website and submitted to obtain the probability of whether the molecule contains a green odor or not (Figure 9).

## 3. Discussion

### 3.1. Analysis of the Chemical Properties of Four Representative Groups of Green Odor Molecules

The group 2_Methyl_4_phenyl_2_butanol is an alcohol that is enough a part of the group to take part in acid-base reactions, hydration processes, and several other common alcohol reactions. Because of the hydroxyl group and benzene ring, it has specific solubility and aromaticity. An organic molecule with a thiazole ring, the 4,5-Dimethylthiazolel group, may be engaged in various electrophilic substitution processes and other related reactions. It may also play a role in organic reactions of an aromatic type. Carbonyl functional groups are found in a class of chemical molecules called 6-methyl-3-hepten-2-one group. A carbonyl functional group, or C=O bond, is present in these compounds and is found on the second carbon. The polar functional group carbonyl modifies the molecule’s chemistry, enabling it to engage in both electrophilic and nucleophilic processes. This class of unsaturated compounds may display a variety of unsaturated nature-related traits because of the existence of a carbon-carbon double bond. All compounds containing the hexyl valerate group have an ester functional group—that is, an alkyl group and a carboxyl group (-COO-). An alcohol reacts with a carboxylic acid to produce esters. The hexyl group, an alkyl group with six carbons, is present in these compounds. The solubility and boiling point of the molecule may be impacted by the existence of the branched chain. Despite not being alcohols, these ester molecules may have certain characteristics in common with alcohols due to the presence of carboxyl and alkyl groups. For instance, under the right circumstances, the ester may hydrolyze.

### 3.2. Analysis of the Results of the Docking Study

Four representative groups of molecules were molecularly docked to the receptor proteins and better results were obtained. Each group of docked small molecules generates strong hydrogen bonding interactions with the target protein. A variety of intermolecular interactions such as van der Waals forces, π-alkyl interactions, and other interactions also exist. They further increase the binding stability of the small molecules in the active pocket. All these interactions suggest that the green molecules have stable binding to olfactory receptors. These small molecules are likely to activate olfactory receptors, which in turn act as pheromones.

The study analyzed the pattern of interaction produced by OBD with green odorants. The involvement of different residues in the binding was compared. Among them, TRP114 tends to generate a large number of hydrogen bonds with small molecules, and the binding of green odorants to the OBD protein receptor is significantly affected by TRP114.

The study mapped the HOMO and LUMO orbitals of representative molecules, revealing the active chemical structures of green odorant small molecules. Dimethylthiazolel and 2_Methyl_4_phenyl_2_butanol mainly have their HOMO-LUMO orbitals on the pentagonal and hexagonal rings, respectively. The p-orbital electrons on the appropriate rings produce these orbitals, which are called π and π* orbitals. The carbonyl moiety of molecules 6-Methyl-3-hepten-2-one and Hexyl valerate is the primary focus of the HOMO-LUMO orbitals of these compounds. To be more precise, the lone pair of electrons on the oxygen atom makes up the majority of the HOMO orbital, whereas the p-orbital electrons of the C-O bond create the π* orbital, which is the primary component of the LUMO orbital. With HOMO-LUMO gaps of 5.79 eV and 5.14 eV, respectively, dimethylthiazolel and 6-Methyl-3-hepten-2-one have the smallest of the four, suggesting more reactivity. Conversely, Hexyl valerate and 2_Methyl_4_phenyl_2_butanol display larger gaps, with values of 7.22 eV and 6.59 eV, suggesting lower reactivity.

### 3.3. Analysis of the Results of the Prediction Algorithms

In the prediction program construction, we compared four different machine learning algorithms. MLP scored the highest in all the metrics. The individual metrics of MLP are as follows: Pre value is 0.81, ACC value is 8.12, AP value is 7.61, F1 value is 8.13, MCC value is 6.24 and AUC value is 8.11. Thus, the MLP algorithm was used to write the program and the study also constructed a prediction website based on the MLP algorithm.

In the factor analysis, the study validated nine important green odor molecular features. It can be seen that Factor 2 provides three main qualities including Morgan’s molecular fingerprint, while Factor 1 provides four main attributes including MolWt, Heavy Atom MolWt and four other features. With the help of factor analysis, we identified nine key characteristics. They were further analyzed for differences. The results showed that green and non-green odor molecules differed significantly in terms of their corresponding qualities. The non-green odor molecules have higher PSA and TPSA, whereas the green odor molecules have lower molecular weight, electron number and higher Morgan’s fingerprint 1-3. Green odorants and other molecules tend to have distinguishable differences in these structural features. We hypothesize that these features may be important factors in the production of green odors.

## 4. Materials and Methods

### 4.1. The Workflow of the Study

**Figure 10:**
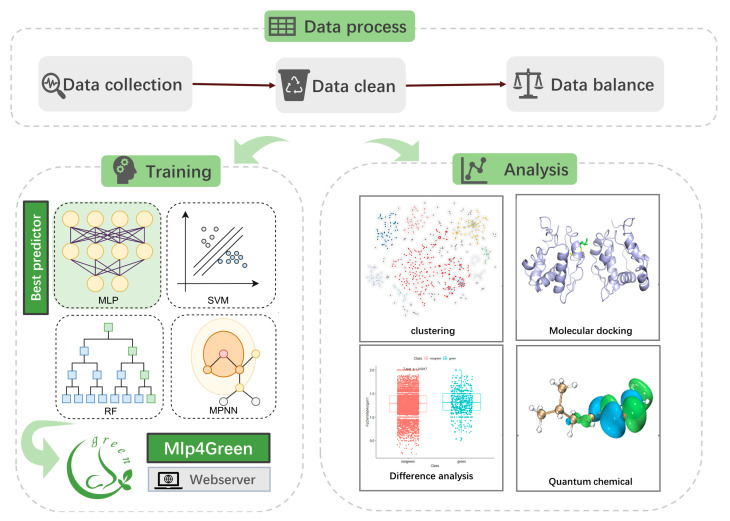
“Mlp4Green” flowchart for predictive analysis of green odors.

In Figure 10, our workflow is displayed. To obtain the training dataset, we extracted the green and non-green odors from the database molecules, cleaned them, and balanced them. Four models (SVM, MLP, RF, and MPNN) were trained on the processed dataset, and the top model was chosen to be implemented on the website. In parallel, molecule docking, quantum chemistry calculations, factor analysis, clustering, and difference analysis were carried out.

### 4.2. The Dataset Processing

The data for the article was downloaded from the LRI & Odour Database—Odour Data, Flavornet and The Good Scents Company Information System, as well as the paper by Hatanaka et al. [2,21]. We extracted 9944 non-green odor molecules and 642 green odor molecules from the database, as indicated in Figure 1. After deleting molecules that RDKit and DGL were unable to identify, as well as duplicates, the data was cleaned, yielding 5758 non-green odor molecules and 587 green odor molecules. In order to achieve a 1:1 ratio of green odor molecules to non-green odor molecules, the 5758 non-green odor molecules were stratified and up-sampled to 588 molecules. A link is provided to the source code of the organized and balanced data: mlp4green/data_clean.py at main—heyigacu/mlp4green—GitHub. Ten repetitions and five cross-validations were performed on the final green/non-green dataset.

### 4.3. The Models for Green Odor Prediction

We used the four models for training green odor predictors, with detailed information on the models below:

(1) We built MLP [22] using PyTorch 2.2.1 (https://pytorch.org/, accessed on 5 December 2023). First, using RDKit 2023.9.5 (https://www.rdkit.org/, accessed on 3 December 2023), the molecules in the dataset were transformed into morgan fingerprints with a radius of 2 and a length of 2048 bits. The batch size was set to 1/16 of the total number of molecules. Subsequently, the fingerprint feature was fed into an MLP, which had two neurons in the output layer, 256 neurons in the hidden layer, and 256 neurons in the input layer. A 0.1 dropout and a ReLU activation function were present between each layer. When the number of times the loss no longer accumulated to seven, the early stop method was utilized to end the training. The trainer employed the cross-entropy loss function, the Adam optimizer, and a learning rate of 0.001.
(2) SVM [23] was built using scikit-learn 1.4.1 (https://scikit-learn.org/, accessed on 1 December 2023). The SVM uses the same input as the MLP mentioned earlier. A 5-fold cross-validated grid search technique was used to identify the SVM’s ideal parameters. The optimal parameters were “C”: 1, “gamma”: 0.1, “kernel”: “rbf”, and “probability”: True.
(3) The RF model [24] uses the same input as the SVM mentioned previously. A 5-fold cross-validated grid search technique was used to identify the RF’s ideal parameters. The optimal parameters were “max_depth”: 6, “max_features”: “log2”, “min_samples_leaf”: 50, “min_samples_split”: 2, “n_estimators”: 100, and “probability”: True.
(4) Using DGL-LifeSci 0.3.1 (https://lifesci.dgl.ai/, accessed on 6 December 2023), we constructed the MPNN [25]. The DGL graphs of the molecules were the MPNN’s input, and a batch size of 1/16 of the total number of molecules was selected. Using canonical atom and bond featurization, node feature and edge feature embedding produced 74 one-hot coding features for atoms and 12 one-hot coding features for bonds. The MPNN’s node output dimension and edge output dimension were both set to 64 and 128 correspondingly, with the remaining parameters remaining at their factory settings. The training parameters, similar to MLP, include early stopping, learning rate, loss function, and optimizer.

### 4.4. Performance Evaluation

Five commonly used indicators were introduced in order to effectively [26], and quantitatively assess each binary classifier model’s performance:

Precision = TP/(TP + FP)(1)

Accuracy = (TP + TN)/(TP + TN + FP + FN)(2)

Recall = TP/(TP + FN)(3)

F1 = 2TP/(2TP + FN + FP)(4)

MCC = (TP × TN − FP × FN)/√((TP + FP)(TP + FN)(TN + FP)(TN + FN))(5)

where the numbers for correctly classified positives, correctly categorized negatives, incorrectly classified negatives, and incorrectly classed positives are represented, respectively, by the symbols TP (True Positives), TN (True Negatives), FP (False Positives), and FN (False Negatives). AUC is the probability that given a random positive sample and a negative sample, classified and predicted with a classifier, the score of that positive sample is greater than the score of that negative sample.

### 4.5. Clustering of the Green Odor Molecules

The morgan fingerprint vector, which had a length of 2048 and a molecule radius, was computed using RDKit. The dimensionality was then reduced to two dimensions using t-distributed Stochastic Neighbor Embedding (t-SNE) [27]. The odor molecules’ chemical space is represented by this space. We selected a new clustering technique that can differentiate the edges of distinct classes more effectively than the K-means method. This approach visits all the molecules to finish the clustering, considering two molecules as a group if their distance from one another is less than 1/24 of the distance between the two furthest molecules in the full collection of molecules. Using Matplotlib 3.8.3 (https://matplotlib.org/, accessed on 9 December 2023), we visualized the clustered chemical spatial network (CSN). This involved displaying the node radius, which represents the distance cutoff, and the edge thickness, which represents the dice similarity between two molecules. You may obtain the aforementioned clustering code from https://github.com/heyigacu/DistanceClustering (accessed on 11 December 2023).

### 4.6. Chemical Space Network Mapping

Our study created a map of the chemical space network (CSN) using the similarity between chemical fingerprints [28,29]. Every molecule has a specific position in a theoretical region called chemical space. The closer the molecules are in place, the more similar their characteristics and properties are. CSN [30] is one tool for analyzing and displaying interactions in small molecule datasets. CSNs are meant to provide an alternate representation to coordinate-based visualization by utilizing molecular descriptors. In a CSN, compounds are usually shown as nodes connected to edges, where an edge is a connection between two compounds. We used the t-SNE dimensionality reduction approach to obtain each odor compound node coordinates.

### 4.7. Factor Analysis and Difference Analysis

For factor analysis, we chose 208 molecular property descriptors from the computational chemistry software RDKit 2023.9.5 (https://www.rdkit.org, accessed on 12 December 2023). The component matrix was generated by choosing the right number of common elements, and the component matrix score was computed after passing the KMO and Bartlett’s spherical test [31]. Selecting descriptors for the component matrix that had factor loading values higher than 0.75 allowed us to determine if the distributions of A and B differed noticeably. If you want to determine whether there is a significant difference between the two samples, perform the Shapiro-Wilk test [32] first. If not, use the Mann-Whitney U test [32]. To find out if there was a difference between the two samples, we utilized the Welch *t*-test [33] when the variance was not homogenous and an independent *t*-test otherwise. All of the paper’s significant difference tests used the test analysis described above (https://scipy.org/, accessed on 11 December 2023). They were carried out with a *p*-value of less than 0.01.

### 4.8. Dice Coefficients to Compute Morgan Fingerprint Similarity

Dice is a centralized similarity measure that is commonly used to calculate the similarity between samples [34]. The formula is:

DSC(A,B) = (2 × |X∩Y|)/(|X| + |Y|)(6)

Morgan fingerprinting [35], sometimes called circular fingerprinting, is a feature representation used in cheminformatics to characterize molecules. Morgan fingerprinting generates bit vector representations by taking into account the local environment of the molecule, and these bit vectors are commonly used in applications such as molecular similarity searches and virtual screening. We used the dice coefficient for each group of green odor molecules to determine the similarity between their morgan fingerprints. For each group of green odor molecules, we chose the molecule with the highest similarity score as the representative molecule of the group [36] (Table 1).

### 4.9. Molecular Docking and Quantum Chemistry Calculations

We selected representative molecules from each of the four groups of green odorant molecules as ligands. The structure and active site information of the Anopheles gambiae odor-binding protein 1 have been extensively studied. Green odor can attract or repel insects. Insects can communicate, alert, and attack by inhaling green odor as a pheromone. Green odor is closely related to the olfactory connection of insects. In this study, we used Anopheles gambiae odor-binding protein 1 as an olfactory receptor to investigate the mechanism of green odor production. The structure files were obtained from the PDB database (PDB ID: 3N7H [37]). The original structure files were dewatered and other ions were removed. Molecular docking was performed using AutoDock Vina 1.2.5 [38]. We used Gaussian 09 (https://gaussian.com, accessed on 15 December 2023) to perform quantum chemistry calculations [39] for the above molecules, and Multiwfn to visualize the LUMO and HOMO orbitals.

### 4.10. Webserver

The front-end of the website used the front-end language React v18.0 (https://react.dev/, accessed on 16 December 2023) and user interface (UI) library Antd 5.x (https://ant.design, accessed on 16 December 2023), while the back-end used Django 5.0 (https://www.djangoproject.com/, accessed on 16 December 2023) based on the model-view-controller (MVC) framework, and the server was real-time responsive.

## 5. Conclusions

In this study, we constructed a green/non-green odor determination procedure based on the MLP algorithm and created a website https://hwwlab.com/webserver/mlp4green, (accessed on 20 December 2023). It successfully predicts whether a molecule contains a green odor with 81.2% accuracy and high levels of recall, f1-score, and other performance metrics. In the study, 587 green odor molecules were analyzed by clustering and molecular docking. Compared to non-green odor molecules, green odor molecules are smaller in terms of molecular mass, FpDensityMorgan, and electrons. LEU 76, ALA 88, MET 89, and TRP 114 play important roles in the green odor mechanism of action. The full code for this study is publicly available at https://github.com/heyigacu/mlp4green, (accessed on 20 December 2023).

This study confirms the hypothesis that there are common features in the structure of green odor molecules and it successfully constructs a prediction method with high accuracy. The good results of molecular docking also reveal the possible mechanism of green odor molecules playing the role of insect pheromone, which supports the validity of the prediction tool. Existing experimental screening methods are difficult to achieve efficient and economical discovery of new molecules in the face of the huge amount of plant volatile compounds. With the rapid development of artificial intelligence, the first incorporation of deep learning methods in the study of green odor molecules can be regarded as a sign of a new era in which green odor research has stepped into intelligence and standardization.

However, due to the numerous roles of green odor molecules, there are many potential possibilities for their mechanism of action. In this study, only the aspect of attracting or repelling insects among them was chosen as an example to explore. Meanwhile, due to the fact that deep learning itself is characterized by high accuracy but low interpretability, this study did not derive the most essential structural features of green odor molecules, but simplified the research system by clustering, mainly analyzing the representative molecules of each cluster.

In order to clarify the target proteins of green odor molecules in multiple biological pathways, subsequent studies can be considered for the analysis of antidepresssant-related pathways and experiments on antibacterial mechanisms. Relevant receptors in humans or mice can also be selected for molecular docking studies. After the target proteins are experimentally confirmed, molecular dynamics simulations can be performed to explore the differences in the mechanism of action of different green odor molecules. The next step of the machine learning study can be directed to the extraction of green molecular features captured by the computer.

The prediction tool constructed in this study facilitates the discovery of new green molecules. The library of green odor molecules, as a potential source of computer-aided drug design, is likely to be expanded relatively quickly by this. The research process of clustering-machine learning prediction-difference analysis-molecular docking in this study is likely to be further used for property exploration and prediction studies of other bioactive compounds.

## Figures and Tables

**Table 1:** The four most numerous groups after clustering and their representative molecule.

Group	Molecule Name	Closeness Centrality
4,5-Dimethylthiazole Group	4,5-Dimethylthiazole	0.16
6-Methyl-3-hepten-2-one Group	6-Methyl-3-hepten-2-one	0.07
Hexyl valerate Group	Hexyl valerate	0.16
2-Methyl-4-phenyl-2-butanol Group	2-Methyl-4-phenyl-2-butanol	0.20

**Table 2:** Component Matrix Sorted and Filtered.

Features	F1	F2	F3
MolWt	0.97	−0.15	0.19
FpDensityMorgan1	−0.41	0.85	0.06
FpDensityMorgan2	−0.23	0.97	−0.03
FpDensityMorgan3	0.06	0.95	−0.11
HeavyAtomMolWt	0.96	−0.14	0.23
NumValenceElectrons	0.97	−0.16	0.16
TPSA	0.21	−0.04	0.98
pyLabuteASA	0.98	−0.15	0.11
PSA	0.21	−0.04	0.98

## Data Availability

Data are contained within the article.
